# 

**Published:** 1883-04

**Authors:** 


### Change of Time.

At a meeting of the Executive Committee recently held, it was decided to hold the next annual meeting of the California State Dental Association on Tuesday, May 22, instead of the first Tuesday of June, as designated at the meeting last year.

S. E. GOE, Sec.

### Ohio College of Dental Surgery.

The thirty-seventh annual commencement of this institution was held in College Hall, Cincinnati, Wednesday evening, March 7, 1883, at 8 o'clock.

The annual address was delivered by Prof. L. Buffatt, M.D., D.D.S., of Cleveland, O. And the address on behalf of the class by J. M. Letcher, jr., D.D.S.

### N. O. D. A.

The twenty-fourth annual meeting of the Northern Ohio Dental Association will be held in the city of Sandusky on Tuesday and Wednesday, May 8 and 9, beginning at 10 o'clock A.M.

The meeting will be held in the parlors of the Sloan House. A cordial invitation is extended to all the profession, and it is greatly desired that there be a large attendance. The following subjects will be presented for discussion, viz.:

1. Methods and Materials for Filling Teeth.
2. Diseases of the Gums; Causes and Treatment.
3. Should the Operative and Prosthetic Departments of the Dental Art be separated?
4. Pulpitis.
5. Miscellaneous Business.

### Removal.

Within the last few weeks Dr. W. Storer How, who for some fifteen years or more has been a successful dental practitioner in Cincinnati, has abandoned his practice here and taken up his abode in Philadelphia.

The Doctor is a Christian gentleman with all that term means; he is a man whom society and the public can ill afford to lose. The profession, especially, of which he was a member in this city will feel his loss greatly, as he was ever active for its interests, and did much to promote its progress and elevation.

He has not wholly severed his connection with the profession, but is directing his efforts and energy in a channel aside from the regular practice and one which may be of more extended usefulness.

The profession of this city cannot afford to lose such men from its ranks, but the loss here is gain somewhere else. The kind remembrances and best wishes of a host of friends will follow—yes, accompany—the Doctor and his estimable wife wherever they may go.

CHICAGO, April 10, 1883.

EDITOR REGISTER.--The Chicago Dental Society elected the following officers for the ensuing year on April 3:

President, C. P. Pruyne; 1st Vice-President, C. F. Matteson, D.D.S.; 2d Vice-President, J. W. Wassall, D.D S.; Recording Secretary, R. H. Kimball, D.D.S.; Corresponding Secretary, A. W. Harlan, D.D.S.; Treasurer, E. D. Swain, D.D.S.; Librarian, Jos. G. Reid, D.D.S.; Directors, Geo. N. Cushing, D.D.S., E. Noyes, D.D.S., J. N. Crouse, D.D.S.

Respectfully, A. W. HARLAN.

### Mad-River Dental Society.

The annual meeting of the Mad-River Dental Society will be held at the Phillips House, Dayton, O., on Tuesday, May 22, at 10 o'clock A.M., and hold the sessions during the day and evening.

The following subjects will be presented for consideration:

1. Prosthetic Dentistry, with special reference to Restoration of Features. Essayists: Drs. C. Bradley and A. Sample.

2. The Restoration of Crowns upon Natural Roots. Essayists: Drs. A. T. Whiteside and C. I. Keely.

3. Treatment of Irregularities, and the proper Antagonisms of Natural and Artificial Teeth. Essayists: Drs. Geo. W. Keely and B. Corson.

4. What should be done further to Protect the Public from the increasing impiricism practiced in our large cities. Essayists: Drs. J. Taft and C. M. Wright.

This society was revived last October, and exhibited such vigor as to greatly encourage its friends and the profession of Mad-River Valley.

The approaching meeting will certainly be one of much interest and profit. A general invitation is extended te all who can, to be present, and come prepared to take part. Every dentist owes it to himself, and to the profession, to give as well as to receive.

### CONTENTS.

COMMUNICATIONS.

### THE DENTAL REGISTER,

A MONTHLY MAGAZINE DEVOTED TO THE INTERESTS OF THE

### DENTAL PROFESSION.

*Terms Reduced to* $2.00 *Per Year. Single Copies*, 25 *Cents.*

### EDITED BY PROF. J. TAFT, D.D.S,

—AND—

### *Published by SPENCER & CROCKER, Ohio Dental Depot.*

The volume commences in January and closes in December.

The REGISTER being issued monthly is always fresh, therefore Indispensable to the practitioner who desires to keep posted up with the new inventions and improvements which are daily developed. Neither expense nor effort will be spared to give value and variety to its contents. Articles will be illustrated with well executed engravings whenever they will add to the interest or assist to explanation.

Its extensive circulation and frequency of issue make it one of the BEST DENTAL ADVERTISING MEDIUMS in the United States.

All subscriptions must begin with January or July.

Local or special notices, five lines or less, will be inserted for $3 each insertion ; over five lines, 50 cts. per line.

All advertising bills payable in advance, or at furthest, after the issue of the first number containing the advertisement. All transient advertisements to be paid for in advance.

☞ CONTRIBUTIONS SOLICITED.

Exclusively Editorial Communications should be addressed to the Editor.

The latest number of the REGISTER may be ordered with or without other goods at any time, and all letters should be addressed to

**SPENCER & CROCKER, Ohio Dental Depot, Cincinnati, O.**

